# Differences in play can illuminate differences in affiliation: A comparative study on chimpanzees and gorillas

**DOI:** 10.1371/journal.pone.0193096

**Published:** 2018-03-07

**Authors:** Giada Cordoni, Ivan Norscia, Maria Bobbio, Elisabetta Palagi

**Affiliations:** 1 Museo di Storia Naturale, Università di Pisa, Calci (Pisa), Italy; 2 Department of Life Sciences and System Biology, University of Torino, Torino, Italy; University of Portsmouth, UNITED KINGDOM

## Abstract

Play behaviour reinforces social affiliation in several primate species, including humans. Via a comparative approach, we tested the hypothesis that play dynamics in a group of lowland gorillas (*Gorilla gorilla gorilla*) are different from those in a group of chimpanzees (*Pan troglodytes*) as a reflection of their difference in social affiliation and agonistic support. We selected one group of lowland gorillas and one of chimpanzees, hosted at the ZooParc de Beauval (France), managed in a similar way and living in similar enclosures. The same observers video-collected and analysed data on play behaviour in both groups, by applying identical methodological procedures. Data showed that adult play was less frequent in the group of gorillas compare to chimpanzees. Polyadic play, which involves more than two players and is characterised by the most uncertain outcome, was also less frequent in gorillas than chimpanzees. Play sessions were more unbalanced (more unidirectional patterns by one of the player towards the other) in chimpanzees than in gorillas but in the latter play escalated more frequently into serious aggression. Play asymmetry in the gorilla group increased as the number of players increased, which explains why gorillas limited their polyadic playful interactions. In conclusion, our findings on the study groups of apes can be a valuable starting point to expand the study of social play in the great apes to evaluate if inter-individual affiliative relationships really account for the differences in play distribution and dynamics.

## Introduction

Compared to ‘serious’ behaviors, whose functions are immediately evident (e.g., sexual behavior, aggressive behavior), play is a difficult behavior to contextualize from both a functional and an operational point of view (for an extensive definition of play see [1]). When we talk about play we immediately think about its long-term benefits, such as motor, cognitive and social skill improvement [2,3]. However, play has also short-term benefits that are not always obvious to the observer. It has been demonstrated that play can reduce social anxiety linked to particular contexts such as crowded condition (gorillas, [4]; bonobos, [5]), pre-feeding competition (chimpanzees, [6]; bonobos, [7]; common marmosets, [8]; wolves, [9]), intra-sexual (sifaka, [10]) and inter-sexual competition (brown bears, [11]).

In species that are characterized by prolonged immaturity and extended parental care [2], play starts in infancy, peaks during juvenility and decreases at puberty (rodents, [12]; lemurs, [13]; macaques, [14]; chimpanzees, humans, [15]; humans, [16]). In many large-brained mammals, including humans, individuals can acquire information about themselves and conspecifics by playing [17–20]. Recent findings on the distribution of play as a function of sex, age, relationship quality, and distribution of power, suggest that play can be shaped according to the social structure and the inter-individual relationships that characterize each group [21].

A play session is the outcome of cooperative and competitive elements that can be measured and quantified [22,23]. The way the session is built up is predictive of its function (for review [12]). If play is fair and cooperative, it can serve to establish social relationships; on the contrary, if play is highly unbalanced and competitive, it will be used to improve ranking status [3,17]. The short-term adaptive functions of play can be related to the level of cooperation and tolerance of the species considered [24]. For example, adult play rates generally covary with the level of tolerance and social affiliation characterizing the group (ungulates, [25]; rodents, [26]; canids, [22,27]; primates, [28,29]). Inter-individual tolerance favors the retention of play also during the adult phase, thus suggesting that this behavior can provide benefits also during adulthood [10,30]. In particular, play between adults and unrelated juveniles, in its polyadic version (i.e., more than two players [31]) can be used as a "social bridge" strategy to expand the social network of adults [32,33]. Indeed, play has proved a reliable tool to strengthen social bonding, especially in those species that are highly cohesive and cooperative (geladas, *Theropithecus gelada*, [28]; chimpanzees, *Pan troglodytes*, [34]; bonobos, *Pan paniscus*, [30,31,35]).

Despite their phylogenetic closeness, similar cognitive abilities and prolonged immature phase [36–38], chimpanzees and lowland gorillas differ in their social organization.

Chimpanzees live in the so called fission-fusion society formed by several reproductive males and adult females with their offspring [37] that distribute according to fruit availability [39]. Males are philopatric, mostly kin-related [37,40] and, consistently, highly sociable and cooperative; they cement their relationships via grooming, sharing food and supporting each-other during aggressive encounters as well as in territorial defense [41]. In some wild communities and in captivity, a certain level of sociality can be also found between females who engage in grooming sessions and agonistic support and establish long-term relationships [41–43]. In general, grooming (and other social interactions, e.g. body contact, social support) is frequent in both wild and captive chimpanzees [44–48], which can use it as a social investment strategy also to gain reproductive advantages [49].

Western lowland gorillas (*Gorilla gorilla gorilla*) live in areas where fruit is readily available and, as a result, have a more frugivorous diet compared to mountain gorillas (*Gorilla beringei beringei*) [50–53]. Western lowland gorillas live in breeding groups that usually comprise one adult male (silverback), several adult females and immature offspring [54–57]. Both males and females transfer from their natal groups [57,58]. In the wild, female gorillas must associate with a silverback male primarily to avoid infanticide [57–59]. Yet, the vulnerability to large terrestrial predators, such as leopards, can also lead to male-female spatial association [60,61]. Following the death of the leading male, groups typically disintegrate and females seek the protection of a new silverback male by joining new groups [62]. Hence, the spatial proximity to the silverback indirectly leads to a spatial proximity between adult females who rarely interact in active way [59]. Indeed, affiliative interactions, such as grooming, are rare [59]. For example, in a study on a wild population of lowland gorillas, Masi and colleagues [63] never observed grooming except for mothers who occasionally groomed infants less than 2 years old. Even though the authors found that grooming time was determined by group size and other socio-ecological factors.

As suggested by Dunbar [64,65], inter-individual relationships established through social grooming have lifelong consequences for primates. The level of grooming investment and its reciprocity, together with other affliative behaviors, are good indicators of social cohesion of a group [66] and of preferential relationships among subjects [67].

The different social profiles of chimpanzees and lowland gorillas make them two valid models to investigate if this difference also reflects into difference in social play behavior.

### Prediction 1

The involvement of adults in playful interactions is sensitive to the degree of tolerance and social cohesion of a given species [10,30,33,68–72]. Adult play is usually inhibited in species that are characterized by both strong adult competition and low levels of social affiliation (e.g., [24,30,73]). If adult play (as grooming [66]) is a form of social investment [12], especially used in some species that rely on reciprocal social support to cope with competitive relationships, we expect that adult chimpanzees play more than adult lowland gorillas (Prediction 1).

### Prediction 2

When more than two animals join the play session (polyadic play), the unpredictability of the event can increase [74,75]. Hence, in case of polyadic interactions animals have to cope with more difficult and risky play sessions [31,76]. Due to the high uncertainty characterizing polyadic play, we expect it to be formed by shorter sessions than dyadic play both in lowland gorillas and chimpanzees (Prediction 2a). The difficulty to manage a session involving more than two players is particularly pronounced in less cohesive species, because subjects engage less frequently in social affiliation and they are therefore less able to use the experience of previous interactions to prolong the session [24,69]. According to the different levels of social affiliation characterizing the two study groups, we expect that chimpanzees engage in higher levels of polyadic play than lowland gorillas (Prediction 2b).

### Prediction 3

During social play, especially play fighting, animals can balance their offensive and defensive manoeuvres to give the possibility to the playmate to counterattack [25,77]. This playful tactic prolongs the session thus making play more successful [21,73,78]. In many species of mammals, individuals that are more dominant and physically powerful generally limit their strength in order to balance the session while playing with subordinates or physically weaker subjects (self-handicapping behaviour; e.g. squirrel monkeys, *Saimiri sciureus*, [79]; rats, *Rattus norvegicus*, [12]; South American sea lions, *Otaria flavescens*, [73]). During play fighting, some behavioral patterns can be categorized as 'offensive' (e.g., pushes, tackles, bites, and chases), whereas some others can be defined as 'defensive' (e.g., shelter, wriggle, flee). The asymmetry of a play session results when one individual actively attains or maintains an offensive position over the other for most of the time [27]. Play symmetry, which implies a high level of cooperation in play [12,27] has not been reported for all of the species in which this aspect has been considered. Recent studies demonstrated that dogs engage in strongly asymmetric playful sessions possibly due to their competitive and, at the same time, flexible social relationships [22,27,80]. The degree of play asymmetry can also be affected by some intrinsic factors such as sex and age of the players, and their relationship quality [12,81]. In *Macaca tonkeana* and *Rattus norvegicus*, for example, play is strongly symmetric and cooperative and it co-varies with the level of tolerance and affiliation of social groups [12,24,69]. If the symmetry of play sessions is linked to the level of affiliative interactions (grooming and contact sitting) and support between subjects, we expect that chimpanzees engage in more symmetric play sessions compared to gorillas (Prediction 3a). Finally, owing to the different social features of gorillas and chimpanzees based on the different level of social tolerance and affiliation, we expect that factors such as age and sex of the players affect the degree of play asymmetry distribution in gorillas more than in chimpanzees (Prediction 3b).

## Methods

### Ethics statement

This study was approved by University of Pisa (Animal Care and Use Board). Since the study was purely observational the committee waived the need for a permit. The study was conducted with no manipulation of animals.

### Subjects and data collection

#### The study groups

The study was carried out on one group of chimpanzees (*Pan troglodytes*) and one group of lowland gorillas (*Gorilla gorilla gorilla*) hosted at the ZooParc de Beauval (St. Aignain sur Cher, France). The composition of study groups is reported in Table 1. We collected data on chimpanzee and gorilla colonies from October till December 2015.

**Table 1:** Composition of the chimpanzee (*Pan troglodytes*) and lowland gorilla (*Gorilla gorilla gorilla*) groups hosted at the Zooparc de Beauval (St. Aignan sur Cher, France). The data refer to the beginning of the study period.

	KINSHIP	SEX	AGE CLASS	YEAR OF BIRTH
	**CHIMPANZEES**			
Joseph (JO)	Sangha’s father	M	Adult	1975, hand reared
Charlotte (CH)	Domi’s mother	F	Adult	1976
Baraka (BA)	……………‥	F	Adult	1979
Julie (JU)	……………‥	F	Adult	1982, hand reared
Bonobo (BO)	SA, WA, LO, YU mother	F	Adult	1982, hand reared
Gypso (GY)	……………‥	F	Adult	1987
Gamin (GA)	LO, YU, LB father	M	Adult	1989, hand reared
Domi (DO)	Charlotte’s daughter	F	Adult	1989
Micheline (MI)	……………‥	F	Adult	1990, hand reared
Sangha (SA)	Bonobo’s daughter	F	Adult	2006
Wamba (WA)	Bonobo’s daughter	F	Juvenile	2008
Tumba (TU)	Domi’s son	M	Juvenile	2009
Lokombè (LO)	Bonobo’s son	M	Infant	2011
Yumbi (YU)	Bonobo’s son	M	Infant	2014
Lobai (LB)	Domi’s son	M	Infant	2014
	**GORILLAS**			
Inge (IN)	……………‥	F	Adult	1980, hand reared
Kabinda (KA)	MA, MP, MY, KH mother	F	Adult	1982
Tamarilla (TA)	Kuimba’s mother	F	Adult	1986, hand reared
Sheila (SH)	SA, MS mother	F	Adult	1991, hand reared
Asato (AS)	KH, MY, MS, MP, KU, SA, MA father	M	Adult	1991
Khala (KH)	Kabinda’s daughter	F	Adult	2007
Mayombè (MY)	Inge’s daughter	F	Adult	2007
Maisha* (MS)	Sheila’s daughter	F	Sub-adult	2008
Mapenzi (MP)	Kabinda’s son	M	Juvenile	2010
Kuimba (KU)	Tamarilla’s daughter	F	Juvenile	2010
Sawa (SA)	Sheila’s daughter	F	Juvenile	2011
Mayelè (MA)	Kabinda’s daughter	F	Infant	2013

*Maisha left the group one week after the beginning of observations

Based on previous literature on gorillas [82–84], we defined the following age categories: infants (0–3.5 years), juveniles (3.5–6 years), subadults/adolescents (6–8 years), adult females (>8 years), blackback males (8–12 years), silverback males (>12 years). Immature subjects are defined as the sum of infants, juveniles, and subadults [82,84].

Based on Nishida et al. [85] we defined the following age categories for chimpanzees: infants (0–4 yr 11 mo old), juveniles (5–8 yr old), subadult/adolescent males (9–15 yr old), subadult/ adolescent females (9–12 yr old), adult males (>16 yr), adult females (> 13 yr). Immature subjects are defined as the sum of infants, juveniles, and subadults.

By comparing the ages (months) between the subjects of the two species (S1 Table), we found no significant difference for either adults (Mann-Whitney exact test: U = 16.00, N_adG_ = 7, N_adC_ = 10, p = 0.066; gorilla mean age ±SE = 278.86 ±50.29; chimpanzee mean age ±SE = 370.30 ±37.72) or immature subjects (U = 10.00, N_immG_ = 5, N_immC_ = 5, p = 0.690; gorilla mean age ±SE = 59.60 ±10.18; chimpanzee mean age ±SE = 50.00 ±14.33).

The two groups occupied similar indoor and outdoor enclosures. The indoor facilities were about 200 square metres each and the outdoor facilities were 2000 square metres each. The gorilla and chimpanzee facilities were also comparable in terms of hiding places (e.g., vegetation, rocks, holes) and resting places (hammocks and platforms). The indoor facilities were equipped with trunks, lianas, ropes, and platforms so that the animals could move freely. All the outdoor facilities were delimited by an artificial moat. The management schedule of chimpanzees and gorillas was the same. Animals received food (fruit, vegetables, yogurt, seeds and grains, branches with green leaves) four times per day approximately at the same hours and they were not separated during the feeding. The food was scattered on the ground. Water was available *ad libitum*. No stereotypic or aberrant behaviours were observed in the two groups.

#### Data collection

The observations took place daily over a 6-h period that spanned morning and afternoon (including feeding times), in both indoor and outdoor facilities. The two observers carried out the observations on the same group at the same time by following a precise time schedule: e.g., morning day 1 chimpanzees; afternoon day 1 gorillas; morning day 2 gorillas; afternoon day 2 chimpanzees.

Before commencing systematic data collection, the two observers underwent a 35 hr training period to become skilled in animal and behavior identification (defensive, offensive and neutral playful patterns, grooming, contact sitting, agonistic interactions and agonistic support). During the training period the observers collected the same data, which were then compared and discussed. For each behavioral category the Cohen’s kappa was: k_defensiveplay_ = 0.86, k_offensiveplay_ = 0.85, k_neutralplay_ = 0.87, k_durationdefensiveplay_ = 0.87, k_durationoffensiveplay_ = 0.85, k_durationneutralplay_ = 0.88, k_grooming_ = 0.93, k_contactsitting_ = 0.96, k_agonisticinteraction_ = 0.91, k_agonisticsupport_ = 0.89 [86]. This reliability procedure was repeated at the beginning of each month of data collection on a sample of about one hour of observation.

Scan animal sampling [87] was applied to gather data on grooming and contact sitting, by speaking into a tape recorder; scans were carried out every 10 minutes when all the animals were perfectly visible. Via this method we gathered 59.5 hrs of observation for chimpanzees and 65.0 hrs for gorillas.

The all occurrences sampling method [87] (70 hours collected on gorillas and 70 hours collected on chimpanzees) was used to record any displacement/avoidance event and overt agonistic contact occurring in each of the two groups [88]. For each agonistic contact, the aggressor was defined as the subject who engaged in charging, chasing, aggressive pulling/pushing, slapping, biting, stamping. The victim was defined as the subject who engaged in submissive and fear behaviours such as crouching, avoiding, fleeing, and screaming. The supporter was defined as the individual who attacked the victim (in case of agonistic support in favor of the aggressor) or the aggressor (in case of agonistic support in favor of the victim). We considered as agonistic support every intervention during an ongoing conflict. Since each subject of the two groups was perfectly recognizable, we audio-taped a verbal record of the identities of the aggressor, the victim and the third party acting as supporter both to the victim and/or to the aggressor (when supporting behavior was present).

We also applied the all occurrences sampling method to record all playful sessions (738 for chimpanzees and 565 for gorillas) that occurred within the observation period. Playful interactions were filmed (Digital videocamera Panasonic HC-V180EG-K Full HD Optical zoom 50x and Sony HDR-PJ240) and subsequently analyzed frame-by-frame using the programs Kinovea 0.8.15 and VLC 2.2.1. For each play session we reported i) player identities (name, sex, age), ii) playful behavioural patterns in sequential order (see Table 2), iii) playful facial expressions emitted by both partners (*Play face*: the mouth is opened with only the lower teeth exposed in a relaxed way; *Full play face*: the mouth is opened with both upper and lower teeth exposed in a relaxed way [31,75]) iv) number of players (*dyadic play*: play session involved only two players; *polyadic play*: play session involved more than two players), and v) Play Duration (PD) in seconds.

**Table 2:** Play behavioral items recorded during the study.

Behavioural patterns	Definitions
Acrobatic play (n)	The individual swings hanging/jumps from a support in a solitary and/or social manner
Airplane (n)	The older individuals holds the playmate with the hands/feet above its head while lying on the ground
Finger/hand in mouth (d)	The individual puts its fingers or hand in the mouth of playmate
Gentle wrestling (o)	The individual (generally infant) kicks and fights with the adult playmate that, in turn, gently grabs it
Give me five (n)	Two playmates are positioned face-to-face and slap each other palms
Head beat (o)	The individual hits with its head the playmate
Peek a boo (n)	The individual hides and suddenly pops out from a shelter
Pirouetting (n)	The individual performs somersaults and pirouettes on itself or hanging from a rope
Play bite (o)	The individual bites the playmate in a non-harmful way
Play brusque rush (o)	The individual jumps with its four limbs on the playmate
Play carrying (n)	The individual dorsally or ventrally carries the playmate (usually younger). It is a behavioral pattern typical of play mothering
Play climb or stand on another (o)	The individual climbs or stands on the playmate’s body independently of the position of the playmate (sitting, lying or standing)
Play confront (o)	The individual grabs the shoulders of the playmate standing face to face in bipedal position, pushing or grabbing
Play drag (o)	The individual hauls the playmate taking it from the limbs
Play eye cover (o)	The individual covers the eyes of the playmate
Play grab (o)	The individual grabs the playmate holding it tightly
Play jump (n/o)	The individual gently jumps alone or on the playmate
Play kick (o)	The individual gently kicks the playmate
Play manipulation (n)	The individual takes and explore an object without using it for any specific goal
Play moon walk (n)	The individual walks backward, generally keeping its eyes fixed on the playmate
Play piggy back ride (o)	The individual is placed astride on the back of the playmate
Play pull (o)	The individual pulls the playmate with hands/feet
Play push (o)	The individual pushes playmate with hands/feet
Play recovering a thing (o)	The individual chases the playmate and attempts to grab object carried by it
Play retrieve (o)	The individual blocks the playmate to prevent its flight
Play roll (n)	The individual turns its body from side to side while supine
Play run (o)	The individual runs alone (solitary play); the individual chases the playmate or flights from it (social play)
Play shake the rope (o)	The individual shakes the rope on which the playmate is hanging
Play shelter (d)	The individual protects itself from playmate slaps, bites, etc. by putting its arms over the head
Play slap (o)	The individual gently slaps any part of the playmate’s body
Play slide down (n)	The individual slides down from hill, tree, rocks or other equipment
Play stamp (o)	The individual jumps on the ground (solitary) or on the playmate with its feet (social) in a repeated way
Play swing (o)	The individual dangles hanging on playmate.
Play tug-of-war (n)	The playmates contend an object and pull it toward themselves
Play turn around (n)	The playmates run/walk around an object
Play walk (n)	The individual follows the playmate
Play wriggle (d)	The individual wriggles to get rid of the grip of the playmate
Rough & Tumble	The playmates play in tight and continuous physical contact by employing patterns typical of real fight such as, bite, kick, slap, stamp, etc. It involves many of the behavioral items described in this table
Somersault (n)	The individual flips over the ground or on vertical supports in solitary or social manner
Swing over someone (n)	The individual dangles over playmate who tries to grab it. Also used as a play invitation.
Tickle (n)	The individual (generally the older) tickles with hands/feet the torso of the playmate

**Notes**: **o** = **offensive** pattern (those attack/pursuit playful patterns giving to one of the players a distinct and clear physical advantage over the partner); **d** = **defensive** pattern (those patterns by which the player tries to cope with attack/pursuit playful patterns performed by the partner, the subject performing the defensive pattern generally attains or maintains a losing position); **n** = **neutral** pattern (those patterns i) not showing any attack/pursuit or losing nature, ii) involving an object, iii) including acrobatic motor actions performed concurrently by the players).

#### Operational definitions

A play session began when one partner invited another individual to play (e.g. play slap, play retrieve, touch and play run; [4,6]) or directed any playful pattern toward it. If the partner ignored the invitation this was not considered as a play session. A session ended when playmates ceased their activities, that is, one of them moved away or a third individual interrupted the previous interaction. If another play session began after a delay of 10 s, that session was counted as new.

As for the definition of polyadic sessions, we used the following criteria. If the individuals A and B were playing and C joined in, the session shifted from dyadic to polyadic and the two sessions were considered as distinct. Similarly, if one of the three animals dropped out, the session shifted into a dyadic session and it was considered as a new session. When at least one of the players changed during a polyadic/dyadic playful interaction, that session was considered as a new session.

The overall play frequency and the frequency of either dyadic or polyadic play, were calculated for each individual as the number of play sessions (per play type) per hour. The mean duration of the polyadic or dyadic play sessions was calculated as the total time spent in polyadic or dyadic play for number of polyadic or dyadic sessions performed.

The *Polyadic Play Index* (PPI) was calculated as the number of polyadic sessions minus the number of dyadic sessions on the total of play sessions. PPI values varied from -1 (all dyadic play) to +1 (all polyadic play).

We calculated an *Agonistic Support Index* (ASI) to establish the frequency of the involvement of third parties (coalitionary support) into the agonistic interactions. This index was calculated as follows: the number of supported agonistic contacts (supported conflicts) minus the number of not supported agonistic contacts (not supported conflicts) on the total of agonistic contacts (total conflicts). The ASI ranges from -1 (completely not supported) to 1 (completely supported).

To quantify the level of play asymmetry (*Play Asymmetry Index*, PAI), we classified the patterns in offensive and defensive behaviors [22,27,73,80] (see Table 2 for the definition of the play behavioral items). We calculated the PAI for each session as follows: the number of “wins” for animal A in a dyad equaled the number of offensive behaviors (see Table 2) by A directed at B plus the number of defensive behaviors (see Table 2) by B directed toward A. B’s “wins” were calculated in the same way. Next, we calculated the proportion of “wins” for A as the number of “wins” for A divided by the number of “wins” for both A and B. We calculated the number of “wins” for B in the same way. We subtracted the “A win ratio” from the “B win ratio” by obtaining a value that represented the measure of the degree of asymmetry [22,80,81]. The PAI ranges from -1 to 1 and is calculated as follows:

$$\begin{array}{l} \left[\left(\text{offensive}\;\text{behavior}\;\text{A}+\text{defensive}\;\text{behavior}\;\text{B}\right)+\left(\text{offensive}\;\text{behavior}\;\text{B}+\text{defensive}\;\text{behavior}\;\text{A}\right)+\text{neutral}\;\text{behavior}\right]/\\ \left[\left(\text{offensive}\;\text{behavior}\;\text{A}+\text{defensive}\;\text{behavior}\;\text{B}\right)-\left(\text{offensive}\;\text{behavior}\;\text{B}+\text{defensive}\;\text{behavior}\;\text{A}\right)\right] \end{array}$$

The neutral behaviors are defined and listed in Table 2.

We calculated the PAI value of each session in which the animals A and B were involved. Then, we calculated the mean value of the PAI distribution of the A-B dyad. In case of polyadic play, we calculated the PAI of each dyad involved in the session as follows a-b-c = a-b; a-c; b-c.

By employing the data collected during the agonistic contacts and displacement/avoidance events between individuals (hourly mean frequency ±SE: gorillas, 0.182 ±0.055; chimpanzees, 0.110 ±0.026) we calculated the Normalized David’s Score (NDS values, Table 3) [89]. Normalized David’s scores (NDS) are scalar values assigned to the individuals and were obtained using a dyadic dominance index (D_ij_) in which the observed proportion of wins (P_ij_) is corrected for the chance occurrence of the observed outcome. In particular, D_ij_ calculates the degree in which individual i dominates individual j relative to the total number of interactions between the individuals i and j. This is calculated with Pij = s_ij_ /n_ij_, where s represents the proportions of wins and n the total number of dominance interactions between individuals i and j. To correct for chance, we used the assumption that the n + 1 possible outcomes of s and n are equally likely, leaving the normalized dyadic dominance index corrected for chance to be: D_ij_ = (s_ij_ + 0.5)/(n_ij_ + 1)^4^. Replacing the normal proportions of winning and losing a conflict with the dyadic dominance index scores enabled us to assess dominance scores independent of group size or variation in number of dyadic interactions [89]. We determined the NDS-based hierarchy by ranking the individuals according to their NDS scalar values. The individual NDS values were used for assessing the absolute NDS differences (ΔNDS) for each playing dyad included in the analysis.

**Table 3:** Description of the variables used in the Linear Mixed Model analysis (LMM) of Play Asymmetry Index (PAI). See the text for the explanation of each single variable.

NAME	TYPE
**DEPENDENT VARIABLES**	
Play Asymmetry Index	Continuous
**FIXED EXPLANATORY VARIABLES**	
Number of players	Nominal (0 = dyadic; 1 polyadic)
SEX (player 1 and 2)	Nominal (0 = male; 1 = female)
Duration of the session (secs)	Continuous
delta NDS (absolute value)	Continuous (NDS_PL1_-NDS_PL2_)
Bonding (grooming plus contact sitting)	Continuous (hourly frequency)
AGE (player 1 and 2)	Continuous (months)
**RANDOM VARIABLES**	
Identity of players	Nominal

#### Statistical analyses

When the analyses were carried out at the individual level, we employed non-parametric statistical tests [90]. The Wilcoxon matched-pair signed-ranks test was used to assess difference between dyadic and polyadic play sessions in terms of frequency and length both in chimpanzees and gorillas. The Mann-Whitney-U test for independent samples was employed to check for possible difference between the two species in agonistic support, affinitive (contact sitting and grooming) and play behaviors. We made use of exact tests according to the threshold values suggested by [91]. All the analyses were two-tailed and were conducted using SPSS 20.0.

In order to evaluate possible difference in Play Asymmetry Index between chimpanzees and gorillas, we carried out an analysis at the dyadic level by applying a Two Independent Randomization test (10,000 shuffles) to reduce errors from non independence of data deriving from the presence of the same individual in different dyads [92]. We used the software Resampling Procedures 1.3 by D. C. Howell (freeware, www.uvm.edu/~dhowall/statPages/Resampling/ResamplingPackage.zip).

We evaluated which factors could explain the distribution of PAI via a linear mixed-model (LMM) analysis. PAI was the dependent variable and the identity of the players was entered as random factor (see Table 3). The fixed factors for the LMM analysis are reported in Table 3.

The PAI values of the two species were normally distributed (Kolmogorov-Smirnov, ns). To be conservative as much as possible, we used robust estimation to handle violations of model assumptions. We tested models for each combination involving the variables of interest (Table 3), spanning from a single-variable model to a model including all the fixed factors (full model). The interactions of the different fixed factors were also tested. To select the best model, we used the Akaike’s corrected information criterion (AIC_C_), which corrects the Akaike’s information criterion (AIC) for small sample sizes [93,94]. As the sample size increases, the AIC_C_ converges to AIC. To measure how much better the best model is compared to the next best models, we calculated the difference (ΔAIC_Ci_) between the AIC_C_ value of the best model and the AIC_C_ value for each of the other models. As suggested by Burnham and Anderson [93] and successively by Symonds and Moussalli [94], models with ΔAIC_Ci_ values less than two are considered to be essentially as good as the best model. Moreover, to assess the relative strength of each candidate model, we employed ΔAIC_Ci_ to calculate the evidence ratio and the Akaike weight (*w*_i_; see Tables 4 and 5). The evidence ratio provides a measure of how much more likely the best model is than the model *i*. The *w*_i_ (ranging from 0 to 1) is the weight of evidence or probability that a given model is the best model, taking into account the data and set of candidate models [93,94].

**Table 4:** Results of the LMM analyses on chimpanzee data (dependent variable = Play Asymmetry Index). The Table shows the Corrected AIC value (AICc), the difference between AICc of each model and the model showing the lowest AICc value (ΔAIC_C_) and the AICc weights (w_i_). Only the models with ΔAICc ≤ 4 are reported.

Models	AICc	ΔAICc	wi	wi%	Wbest/wi
Intercept	730.315	0.000	0,629	62.93	*
Number of players (dyadic/polyadic)	734.010	3.695	0.099	9.919	6.343
deltaNDS	734.172	3.857	0.091	9.147	6.879

**Table 5:** Results of the LMM analyses on gorilla data (dependent variable = Play Asymmetry Index). The Table shows the Corrected AIC value (AICc), the difference between AICc of each model and the model showing the lowest AICc value (ΔAICc) and the AICc weights (w_i_). Only the models with ΔAICc ≤ 4 are reported.

Models	AICc	ΔAICc	wi	wi%	Wbest/wi
Number of players (polyadic/dyadic)	444.008	0	0,619	61.958	*
Number of players (polyadic/dyadic) ΔNDS	445.131	1.123	0.238	23.842	2.599
Intercept	447.797	3.789	0,088	8.806	7.035

## Results

Before testing the predictions relative to play dynamics in the two species, we explored if our study groups actually reflected the social characteristics already reported in literature (see the Introduction) for chimpanzees and gorillas. Specifically, we carried out an analysis by comparing the level of grooming, contact sitting and agonistic support (e.g., coalitions during aggression) between the two study groups.

**Fig 1:**
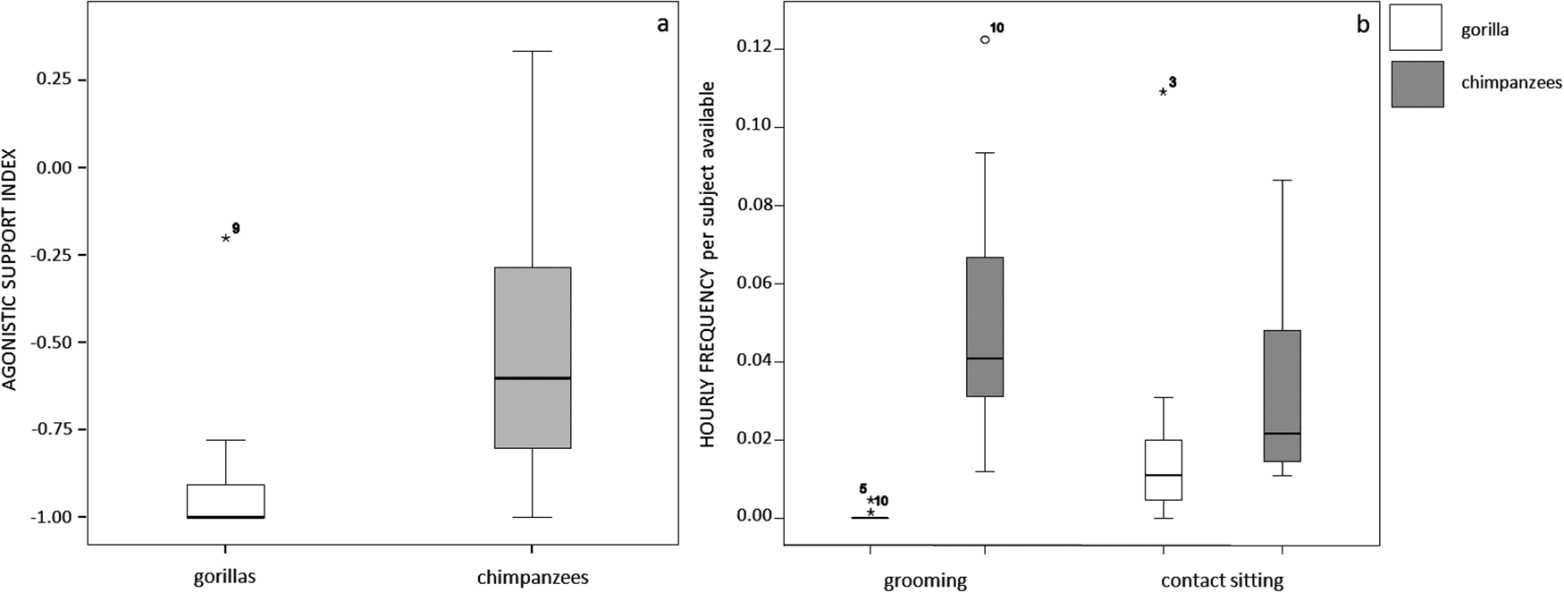
Boxplot showing a comparison of the Agonistic Support Index values between the two study groups (a). Boxplots showing the distribution of the hourly frequency of grooming and contact sitting (per subject available) between gorilla and chimpanzee groups (b). Solid horizontal lines indicate medians; length of the grey boxes corresponds to interquartile range; thin horizontal lines indicate range of observed values.

The values of Agonistic Support Index were significantly higher in chimpanzees than in gorillas (Mann-Whitney exact test: *U* = 27.50, *N*_*G*_ = 10, *N*_*C*_ = 13, *P* = 0.014; Fig 1a; S2 Table). The subjects who were never involved in an agonistic encounter (denominator = 0) were excluded from this analysis.

The hourly frequency of grooming and contact sitting normalized on the number of potential partners strongly differed between the two species. Chimpanzees engaged in higher levels of grooming and contact sitting interactions compared to gorillas (grooming; Mann-Whitney exact test: *U* = 8.50, *N*_*G*_ = 11, *N*_*C*_ = 15, *P* = 0.0001; contact sitting; Mann-Whitney exact test: *U* = 36.00, *N*_*G*_ = 11, *N*_*C*_ = 15, *P* = 0.014; Fig 1b; S3 Table).

### Prediction 1

Hourly play frequency in the gorillas and chimpanzees under study differed according to the age of the players. Play involving the adult subjects of chimpanzees (mean 0.054 ± 0.011SE) was significantly more frequent than play involving the adults of gorillas (mean 0.023 ± 0.015SE) (Mann-Whitney exact test *U* = 14.00; *N*_*AdG*_ = 7; *N*_*AdC*_ = 10; *P* = 0.040). Conversely, play in immature subjects did not differ between the two species (mean_*ImmG*_ 0.479 ± 0.080SE, mean _*ImmC*_ 0.500 ± 0.083SE; Mann-Whitney exact test *U* = 9.00; *N*_*ImmG*_ = 4; *N*_*ImmC*_ = 5; *P* = 0.905). Data are shown in S4 Table.

### Prediction 2

The mean duration of the polyadic play sessions (seconds) was shorter than the mean duration (seconds) of the dyadic sessions in both gorillas (mean duration dyadic play ±SE = 38.25 ±4.02; mean duration polyadic play ±SE = 13.99 ±2.81; Wilcoxon Exact test *T* = 1.00; *ties* = 0; *N* = 8; *P* = 0.016) and chimpanzees (mean duration dyadic play ±SE = 67.89 ±13.07; mean duration polyadic play ±SE = 33.25 ±3.64; Wilcoxon Exact test *T* = 11.00; *ties* = 0; *N* = 15; *P* = 0.003). Data are shown in S5 Table.

**Fig 2:**
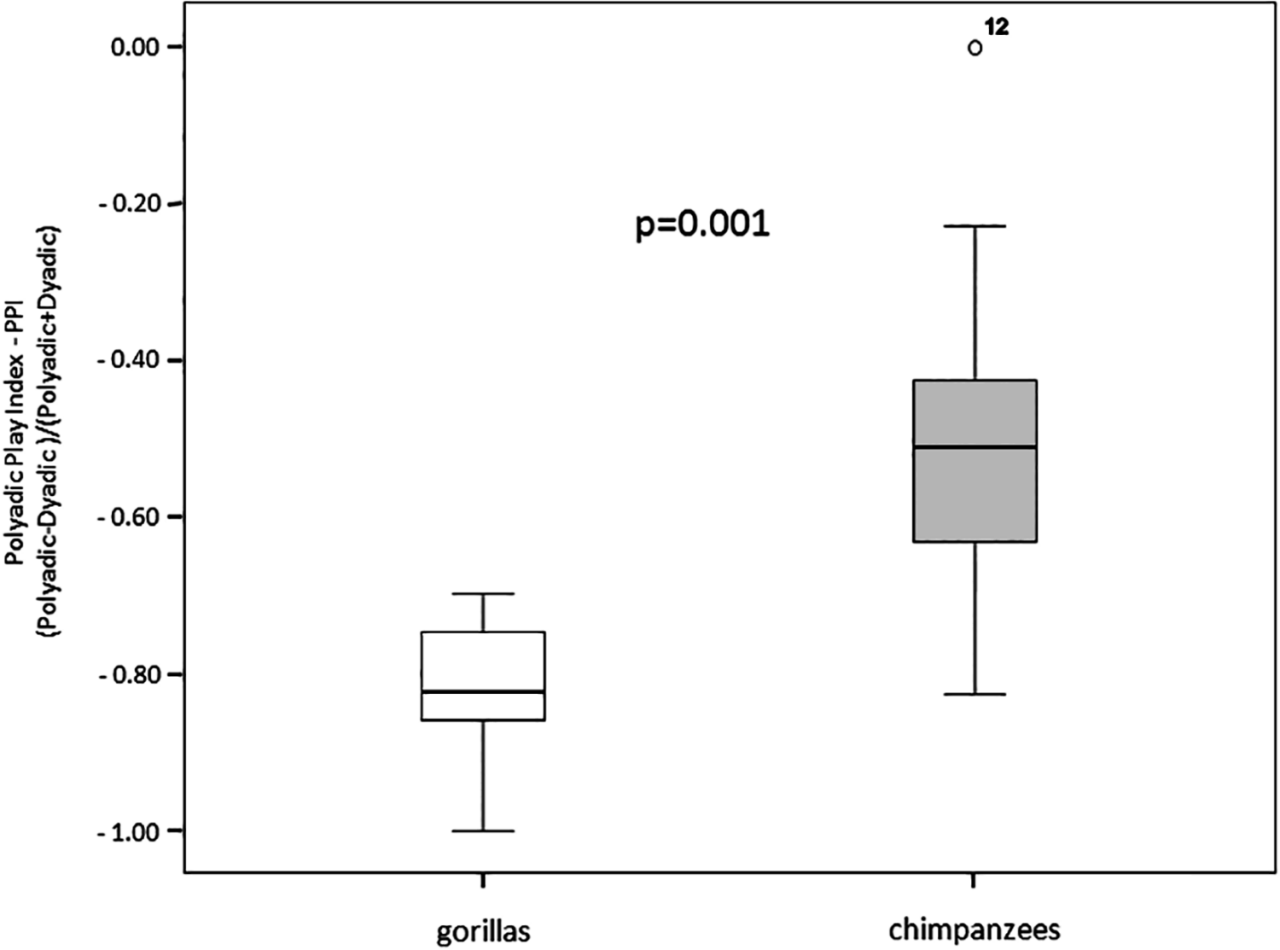
Boxplot showing Polyadic Play Index values recorded in the two study groups.

At intra-specific level, hourly frequency of dyadic play was significantly more frequent than that of polyadic play in both gorillas (Wilcoxon exact test *T* = 1.00, *ties* = 0, *N* = 8, *P* = 0.016) and chimpanzees (Wilcoxon exact test *T* = 11.00, *ties* = 0, N = 15, *P* = 0.003). At inter-specific level the comparison of *Polyadic Play Index* revealed that the relative incidence of polyadic play was significantly higher in chimpanzees than in gorillas (Mann-Whitney exact test *U* = 10.00; *N*_*G*_ = 8; *N*_*C*_ = 15; *P* = 0.001) (Fig 2; S6 Table).

### Prediction 3

**Fig 3:**
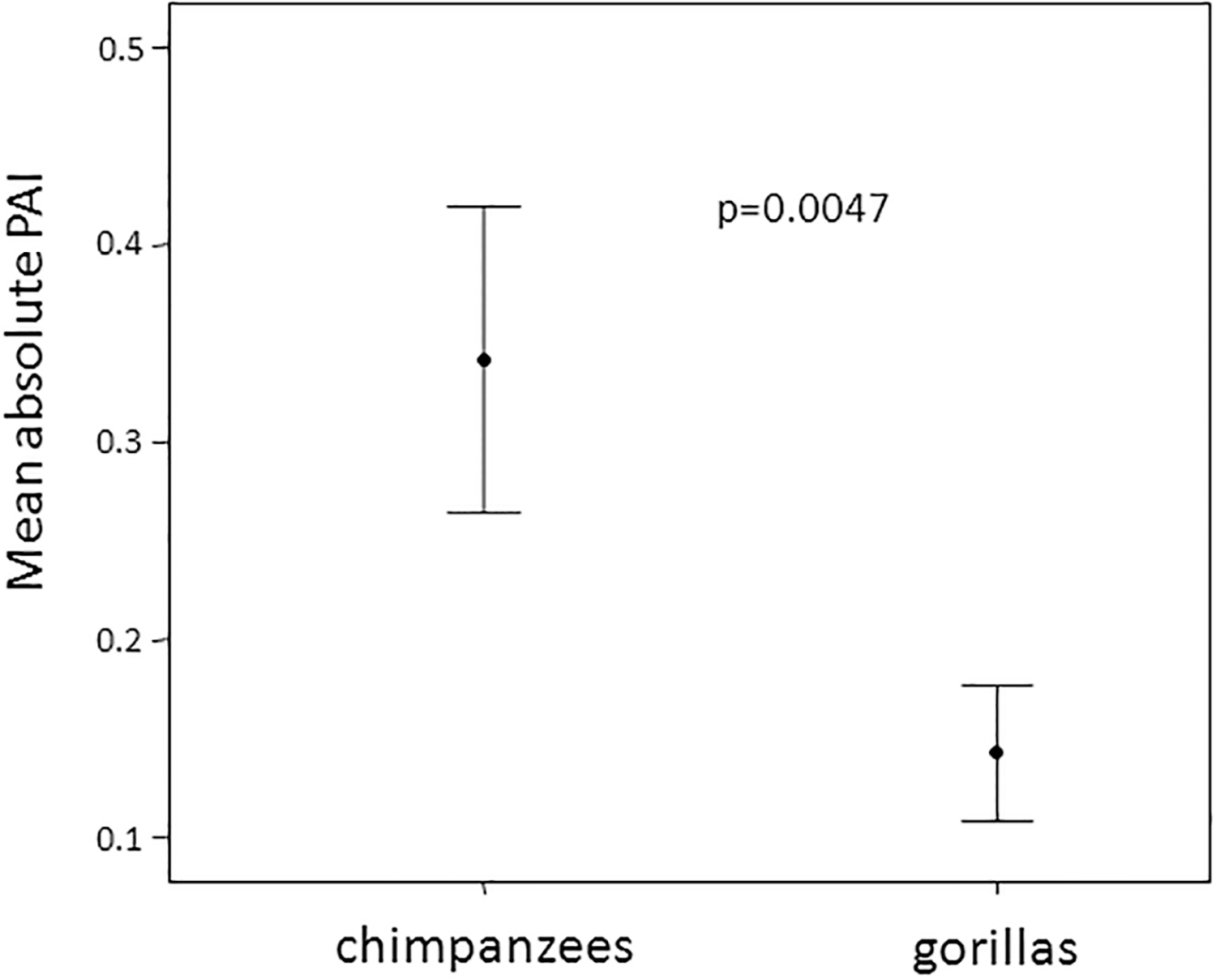
Boxplot showing Play Asymmetry Index values recorded in the two study groups.

To compare the levels of Play Asymmetry Index (PAI) between the two species we calculated the absolute mean value of PAI for each dyad (this analysis was possible only for the dyads which engaged in at least one play session; S7 Table). The Two Independent Randomization test revealed that play in chimpanzees was characterized by higher values of PAI compared to gorillas (*t* = 3.839; *N*_*Gdyads*_ = 16; *N*_*Cdyads*_ = 47; *P* = 0.0047; Fig 3). Using LMM, we assessed which variables explained the distribution of PAI in the two species separately (see Table 3). As for chimpanzees, the best model included the intercept only (*AICc* = 730.315) and had a *w*_*i*_ of 0.629, i.e., there was 62.9% probability that it was the best model. The full model was the worst one (*AICc* = 785.928) (Tables 4 and 6). This result indicates that none of the variables considered significantly affect the distribution of PAI (dependent variable). Data are shown in S8 Table.

**Table 6:** Best LMM models explaining the distribution of Play Asymmetry Index (PAI) in the chimpanzee and gorilla groups.

**Chimpanzees**				
**Fixed variables** (AICc = 730.315)	**Coeff**			**P**
Intercept	0.091			0.013
**Random variables**	**Z**			**P**
Players	2.719			0.007
**Fixed variables** (AICc = 734.010)	**F**	**df1**	**df2**	**P**
Number of players	0.285	1	502	0.594
**Random variables**	**Z**			**P**
Players	2.708			0.007
**Fixed variables** (AICc = 734.172)	**F**	**df1**	**df2**	**P**
delta NDS	0.797	1	502	0.372
**Random variables**	**Z**			**P**
Players	2.692			0.007
**Gorillas**				
**Fixed variables** (AICc = 444.008)	**F**	**df1**	**df2**	**P**
Number of players	6.675	1	316	0.010
**Random variables**	**Z**			**P**
Players	1.375			0.169
**Fixed variables** (AICc = 445.131)	**F**	**df1**	**df2**	**P**
Number of players	8.426	1	315	0.004
deltaNDS	3.257	1	315	0.072
**Random variables**	**Z**			**P**
Players	1.457			0.145
**Fixed variables** (AICc = 447.797)	**Coeff**			**P**
Intercept	0.084			0.02
**Random variables**	**Z**			**P**
Players	1.325			0.185

**Fig 4:**
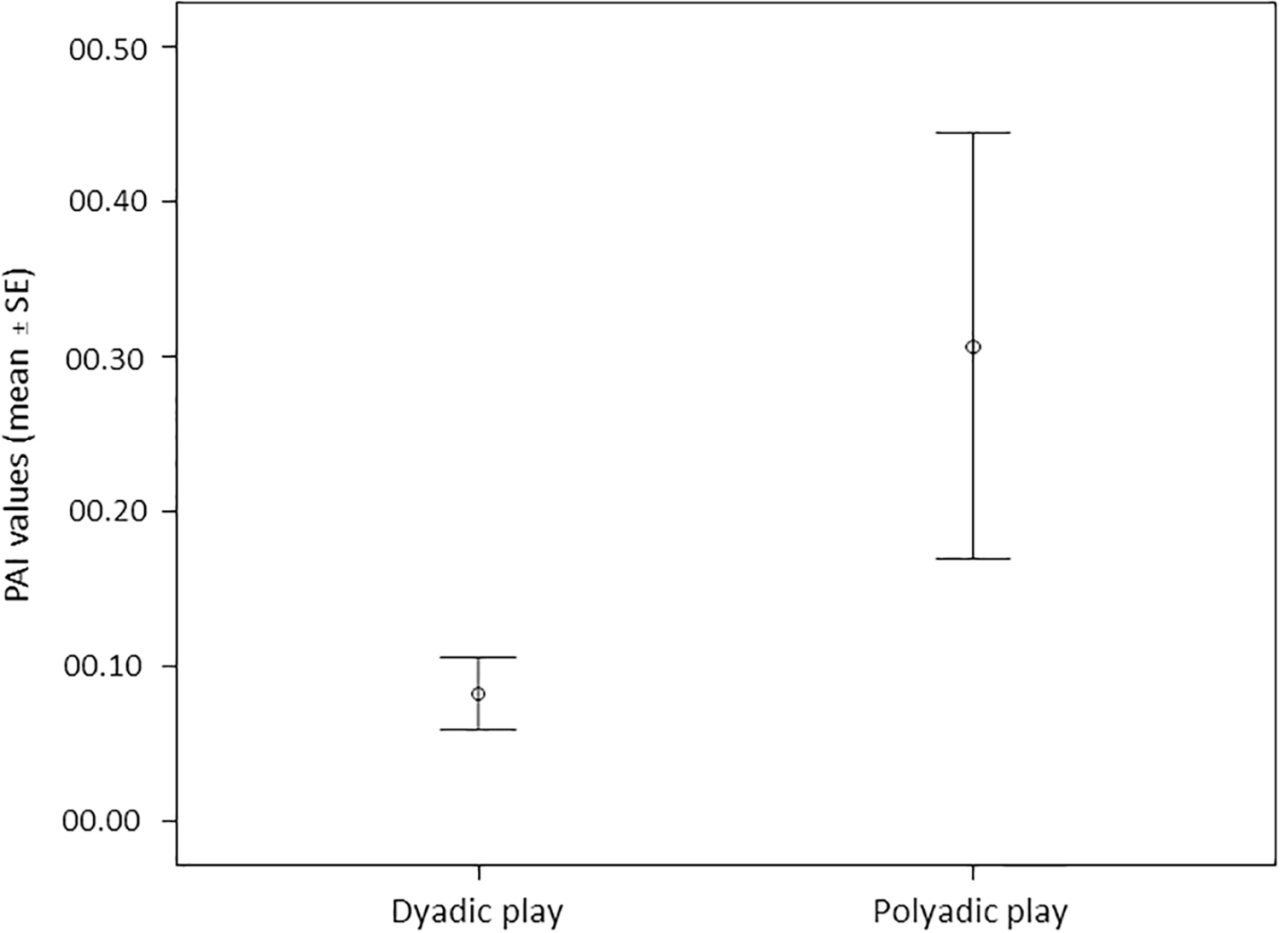
Mean (±SE) of Play Asymmetry Index values relative to dyadic and polyadic play sessions in gorillas.

**Fig 5:**
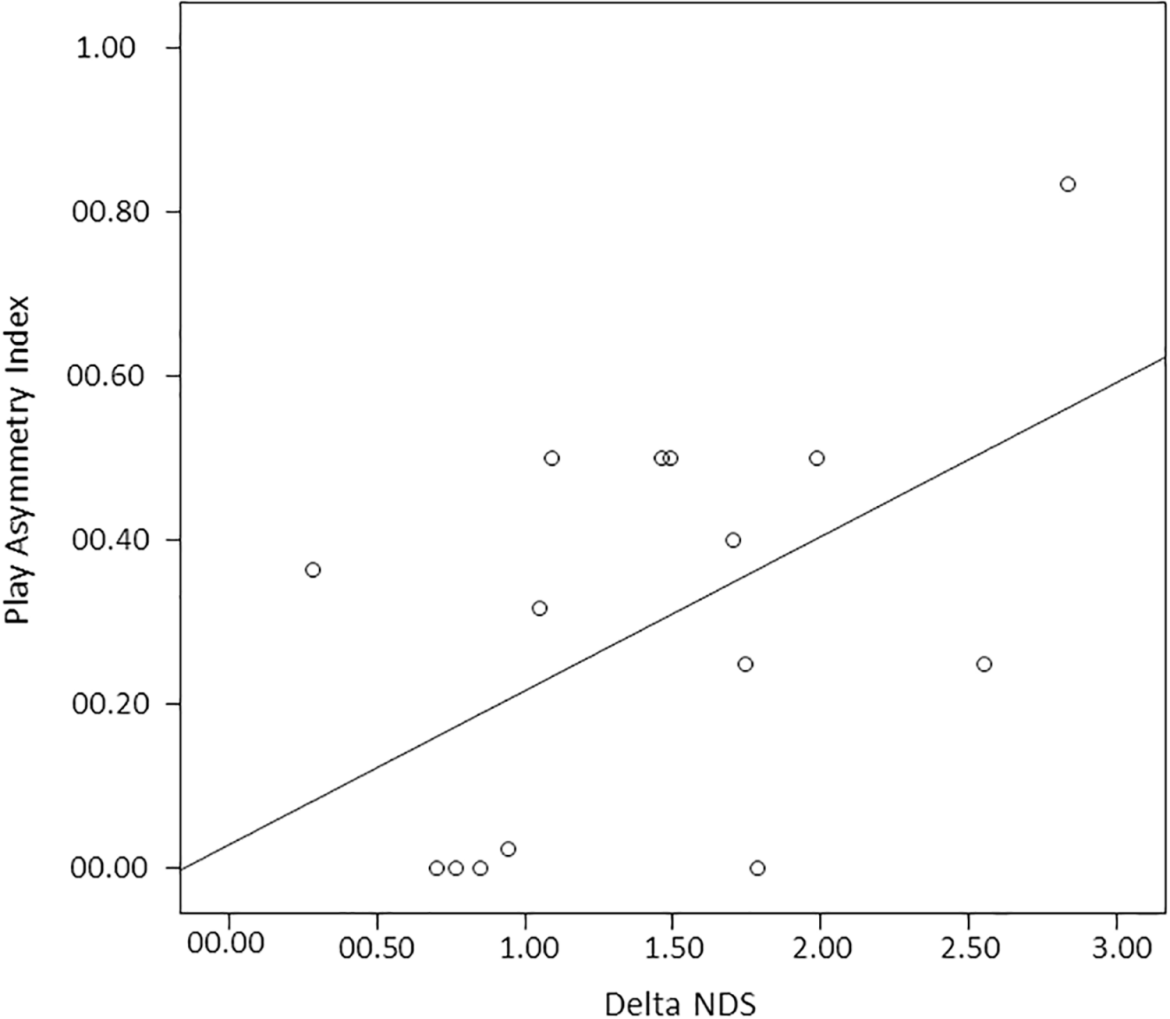
Scatterplot showing the correlation between delta NDS values and Play Asymmetry Index values in gorillas.

As for gorillas, we found that the best model included the number of players only (polyadic *vs* dyadic sessions) (*AICc* = 444.008) and had a *w*_*i*_ of 0.619, i.e., there was 61.9% probability that it was the best model. Nevertheless, the nearest model to the best one included the number of players and ΔNDSs (*AICc* = 445.131) and had a *w*_*i*_ of 0.238, i.e., there was 23.8% of probability that it concurred in describing the distribution of PAI. Because the difference between the two *AICc* values was *<* 2, these two models can be considered as equally valid. The full model was the worst (*AICc* = 496.022). In both the first and the second best models the variable "number of players" had a significant effect on PAI distribution (Tables 5 and 6; Fig 4). This result indicates that the number of players (Fig 4) and the difference in rank (Fig 5) between the players involved had an effect in the distribution of PAI (dependent variable). Play sessions escalated into real aggression more frequently in gorillas (mean 0.031 ± 0.009SE) than in chimpanzees (mean 0.003 ± 0.001SE) (Mann-Whitney exact test U = 31.50; NG = 8; NC = 15; P = 0.046). Data are shown in S9 Table.

## Discussion

The distribution of affiliation and agonistic support in the chimpanzee and gorilla groups under study confirmed that they differed in their social interaction rates. Indeed, our findings showed that chimpanzees engaged in more events of agonistic support (Fig 1a) and spent much more time in close physical contact and grooming interactions compared to the lowland gorillas (Fig 1b; Table 7).

**Table 7:** Behaviors indicating social affiliation and support in chimpanzees and lowland gorillas. The cited literature refers both to wild and captive studies.

	*Gorilla* sp.	*Pan troglodytes* spp.
**Agonistic Support**	Low level^[present study]^	High level^[present study]^
**Grooming**	Low level^[present study,^^96^^,^^97^^]^	High level^[present study,^ ^44^^–^^48^^]^
**Contact sitting**	Low level^[present study,^ ^96^^,^^97^^]^	High level^[present study]^
**Reconciliation**	Limited to alpha male with adult females^[^^98^^,^^99^^,^ ^100^^]^	Involving all subjects^[^^95^^,^^101^^–^^104^^]^
**Consolation**	Only immatures involved as consolers^[^^98^^,^^99^^]^	All subjects involved as consolers^[^^101^^,^^104^^–^^106^^]^
**Play in tense situations (prefeeding/crowding conditions)**	Limited to immatures^[^^4^^]^	All subjects involved^[^^6^^]^

The distribution of affinitive behaviors and coalitionary support obtained for our study groups fits with many previous findings reported in the literature (Table 7). For example, several studies focusing on post-conflict interactions demonstrated that reconciliation, which is a behavioral strategy used to restore the pre-existing social bonding between the aggressor and the victim, is frequent in chimps but not in gorillas (Table 7). Moreover, a similar result has also been reported for consolation, defined as the first spontaneous post-conflict affinitive contact provided by a third party towards the victim of aggression [95].

In wild western lowland gorillas, Stokes [59] recorded low levels of affiliation. In 802 hours of observation, The author reported only six sexual and physical contacts occurring between the silverback and the adult females, whereas no grooming event between adults was observed. Because in western lowland gorillas (as in the other gorilla species) females transfer between groups, the potential benefits from forming long-term alliances may be reduced and, at the same time, the social costs of group change may be low [62]. Moreover, in their study on lowland gorillas of Bai Hokou (Central African Republic), Masi and colleagues [63] reported that the group spent the majority of the time in feeding (67.1%), resting (21.0%) and travelling (11.7%), whereas very little time was dedicated to social activities (0.5%). Even under conditions of limited space availability, captive lowland gorillas showed low levels of social interactions (e.g., social play and physical contact) limiting their levels of proximity as well [107]. In the same conditions, opposite findings were obtained for chimpanzees and bonobos, who did not show significant differences in their levels of social affiliation [5,108,109].

We found that the adult chimpanzees of our group were more frequently involved in play compared to adult gorillas (Prediction 1 supported). It is unlikely that this result could be due to rearing conditions (see Table 1). Clay and coworkers [110] did not find any significant difference in the rates of affinitive behaviors (including social play) between nursery-reared and mother-reared chimpanzees. As for lowland gorillas, Meder [111] showed that when young hand-reared individuals were kept in groups, the rates of social play became comparable to those of mother-reared individuals.

Previous captive and wild studies focusing on play in lowland gorillas did not report a significant amount of play involving adults and sub-adults [63,112–116]. Moreover, Stewart and Harcourt [97] noted that, even if the social behavior most commonly observed in youngster lowland gorillas was play, its rates were inversely related to age just from two years of life. In a study on wild mountain gorillas, Grueter and colleagues [117] found that the consumption of high-quality food (e.g. bamboo shoots) had a positive influence on the level of adult play. Across one year of observation (n_group_ = 3; adult subjects = 32; mean individual observation = 40.4 hours) the mean group frequency of play sessions involving at least one adult was fifty-five. However, the physiological aspects underlying this relatively frequent playful activity need to be investigated more thoroughly. The differences on play distribution we recorded in our study groups cannot be related to the differences in the diets, because both groups were fed with high quality food every day.

A comparison made between the two *Pan* species revealed that adult bonobos (*Pan paniscus*) engaged in higher levels of social play than adult chimpanzees [30,118]. Wobber et al. [119,120] suggested that the maintenance of adult play in bonobos, compared to chimpanzees, is related to their ontogenetic delay in social inhibition. Social inhibition is measured by the low tendency in playing, affiliating, sharing food and cooperating in solving social problems (e.g., agonistic support) [119,120]. To our knowledge there are no experimental data on the development of social inhibition in gorillas to be used to make inferences about the ontogenetic pathways on play dynamics in this species. At this stage, it is however possible to infer that the difference in adult play distribution between the chimpanzee and the gorilla groups is linked to their differences in the levels of affinitive interaction and agonistic support, two proxies of social inhibition [119–120].

The linkage between social affiliation and the distribution of social play is also evident in monkeys. The despotic *Macaca fuscata* shows a strong power asymmetry between dominants and subordinates and low level of agonistic support and social grooming [121–123]. In contrast, the tolerant *Macaca tonkeana* engages in relationships that are minimally influenced by social rank and presents a high proportion of friendly interactions especially among non-kin [124–126]. When comparing the two species, Ciani and colleagues [24] found that *Macaca tonkeana* generally engages in higher levels of play than *Macaca fuscata*. In the two species, play distribution also differed according to the age of the players and their numbers. Compared to Japanese macaques, adult play was more frequent in Tonkean macaques which engaged in higher levels of polyadic play, the most demanding form of social play in terms of physical, cognitive and social skills [69].

In both gorilla and chimpanzee groups, polyadic play sessions had a shorter duration than dyadic ones thus confirming that the increase in the number of players *per se* makes the session more unstable and difficult to manage (Prediction 2a confirmed). In wild chimpanzees, Shimada [127] found that the length of play sessions was higher in dyadic than polyadic cliques and, even when more than two individuals played together, they gradually separated and formed multiple playful pairs. Also our data showed a bias towards dyadic play in both groups, even though the incidence of polyadic play on the total amount of play performed was greater in the chimpanzee than in the gorilla group (Fig 2) (Prediction 2b supported). We also found that, contrary to chimpanzees, in the gorilla group the level of play asymmetry increased as the number of the players increased, possibly indicating the balance of play sessions was destabilized in the lowland gorillas when more than two group mates joined the play session. This interpretation is also supported by the fact that play escalated into real aggression more frequently in gorillas than in chimpanzees. In a previous study on captive chimpanzees, Palagi and Cordoni [118] found that polyadic play frequency negatively correlated with the age of playmates. Our findings on polyadic play in the gorilla group indicate that this behavior is limited from the early stage of life. The difference in the number of adult and immature subjects forming our two groups (Table 1) cannot explain the lower incidence of polyadic play recorded in lowland gorillas as the number of subjects available in each group permitted the formation of triads of playmates. In a study on different groups of Tonkean (number of subjects: group_1_ = 24; group_2_ = 13; group_3_ = 5) and Japanese macaques (number of subjects: group_1_ = 51; group_2_ = 37; group_3_ = 171), Reinhart et al. [69] found that, despite the huge difference in the size of the groups of the two species, the number of players involved in the same session was much higher in Tonkean than in Japanese macaques: all polyadic sessions in Japanese macaques involved only three playmates.

The play sessions recorded in our chimpanzee group were more asymmetric than those of the gorilla group (Prediction 3a not supported). This result could be ascribed to the higher involvement of chimpanzee mismatched dyads (adult-immature pairs). Instead, the LMM revealed that in chimpanzees the Play Asymmetry Index was not affected by any of the variables considered, including the age of the players. Even though some limitations of the methodology used to evaluate the degree of play asymmetry can be present, the greater level of social flexibility makes it possible for chimpanzees to successfully manage unbalanced and unpredictable playful interactions, which rarely escalated into real aggression.

In conclusion, even though this report relies on observation of a single group of lowland gorillas and chimpanzees, our findings are a valuable starting point to expand the study of social play in the great apes in order to evaluate if and how phylogenetic closeness and/or inter-individual affiliative relationships within the groups account for the differences in the distribution and dynamics of the phenomenon.

## Supporting information

S1 Table Dataset of gorillas and chimpanzees including for each individual age categories (ad = adult; subad = subadult; j = juvenile; inf = infant), age in months. (XLSX)

S2 Table Dataset of gorillas and chimpanzees including the number of supported, not supported, total conflicts and Agonistic Support Index (per individual) calculated as follows: (number of supported conflict − number of not supported conflict)/total conflicts. (XLS)

S3 Table Dataset of gorillas and chimpanzees including for each individual grooming and contact sitting hourly frequencies and the hourly frequency of the grooming and contact sitting normalized on the number of potential partners. (XLS)

S4 Table Dataset of gorillas and chimpanzees including play hourly frequency normalized on the number of the potential play partners and age class of individuals (a = adult; j = juvenile; i = infant). (XLSX)

S5 Table Database of gorillas and chimpanzees including the mean duration of dyadic (first column) and polyadic play session (second column) in seconds. (XLS)

S6 Table Dataset of gorillas and chimpanzees including the Polyadic Play Index for each subject included in the study. (XLSX)

S7 Table Dataset of gorilla and chimpanzee dyads including the mean value of Play Asimmetry Index (mean PAI) and absolute value of mean PAI (abs mean PAI) for each dyads. (XLSX)

S8 Table Dataset of gorillas and chimpanzees: dyads (IDplayers), Play Asymmetry Index (PAI), play session duration, dyadic/polyadic play (dia_poli), player sex (sex1, sex2), player age (age1, age2), NDS score difference (deltaNDS), inter-player bonding (bonding). (XLSX)

S9 Table Dataset of gorillas and chimpanzees including for each individual the relative frequency of escalated sessions. (XLSX)
